# The Association between Eating Traits and Weight Change after a Lifestyle Intervention in People with Type 2 Diabetes Mellitus

**DOI:** 10.1155/2018/9264204

**Published:** 2018-06-03

**Authors:** Anitra D. M. Koopman, Maya vd Ven, Joline W. Beulens, Laura M. Welschen, Petra J. Elders, Giel Nijpels, Femke Rutters

**Affiliations:** ^1^Department of Epidemiology and Biostatistics, VU Medical Centre, Amsterdam, Netherlands; ^2^Amsterdam Public Health Research Institute, Amsterdam, Netherlands; ^3^Julius Centre for Health Sciences and Primary Care, University Medical Centre Utrecht, Utrecht, Netherlands; ^4^Department of General Practice, VU Medical Centre, Amsterdam, Netherlands

## Abstract

**Aims:**

To date, studies on the role of eating traits in weight loss success have only included obese people without type 2 diabetes mellitus (T2DM), thereby disregarding negative effects of T2DM-related metabolic changes. Our aim was to assess the association between eating traits and weight change after a lifestyle intervention in people with T2DM.

**Methods:**

For the current study, we reexamined data from a six-month intervention in 120 participants. We determined eating traits at baseline, using the DEBQ, which were used to produce three groups: unsuccessful dietary restrained (high restraint, high emotional/external eating scores), successful dietary restrained (high restraint, low emotional/external eating scores), and reference (low restraint, high or low emotional/external eating scores). Linear regression was used to study the association between the eating trait groups and weight changes after six months, while correcting for possible confounders.

**Results:**

On average, the weight loss success was limited, with a third of the participants being weight stable, a third losing weight > −1 kg (average loss −2.6 ± 1.9 kg), and a third gaining weight > +1 kg (average gain +3.3 ± 1.9 kg). When compared to the reference group, the unsuccessful dietary restrained gained weight during the intervention (beta = 1.2 kg, confidence interval (CI)% = 0.1; 2). No significant change was observed in the succesful dietary restrained group.

**Conclusions:**

The eating trait of being unsuccessfully dietary restrained is associated with weight-loss failure after a six-month lifestyle intervention in people with T2DM.

## 1. Introduction

Moderate weight loss (>5%) improves glycaemic control in people with type 2 diabetes mellitus (T2DM) as well as reduces the risk for cardiovascular disease and mortality [1–5]. However, weight loss in people with T2DM is difficult to achieve [6–8]. Recent meta-analyses of weight loss studies in people with T2DM [9–11] showed that lifestyle interventions result in an average 1–9% weight loss after six months. Studies however often report average weight loss, which omits the fact that some people did lose weight, averaged out with those being weight stable or gaining weight.

Research into factors related to difference in weight loss in overweight people and obese people (all without T2DM) has identified genetic factors, young age, male sex, a high baseline body weight, and older age of obesity onset as predictors [6, 12, 13]. A recent systematic review showed the strong effect of psychological and behavioural factors on weight-loss success [13], with a special focus on eating traits.

A combination of two specific eating traits, namely, high cognitive control over eating (restraint) and low stress-resistant eating (disinhibition), is strongly associated with weight loss success in obese individuals without T2DM, subjected to a lifestyle intervention [12, 14]. We hypothesize that this specific combination of eating traits, namely, high cognitive control over eating (restraint) and low stress-resistant eating (disinhibition), is also strongly associated with weight loss success in people with T2DM.

However, to date, previous studies have only been conducted in people without T2DM and by doing so, they disregard negative effects of T2DM-related metabolic changes. For example, insulin treatment averts weight loss, due to insulin preventing loss of glucose via urine and thereby stimulating fat accumulation [6, 12, 13]. Therefore, the aim of our study was to assess the association between eating traits and weight change after a lifestyle intervention in people with T2DM. We reexamined data from an ineffective lifestyle intervention in people with T2DM [15, 16].

## 2. Methods

### 2.1. Study Design

**Figure 1:**
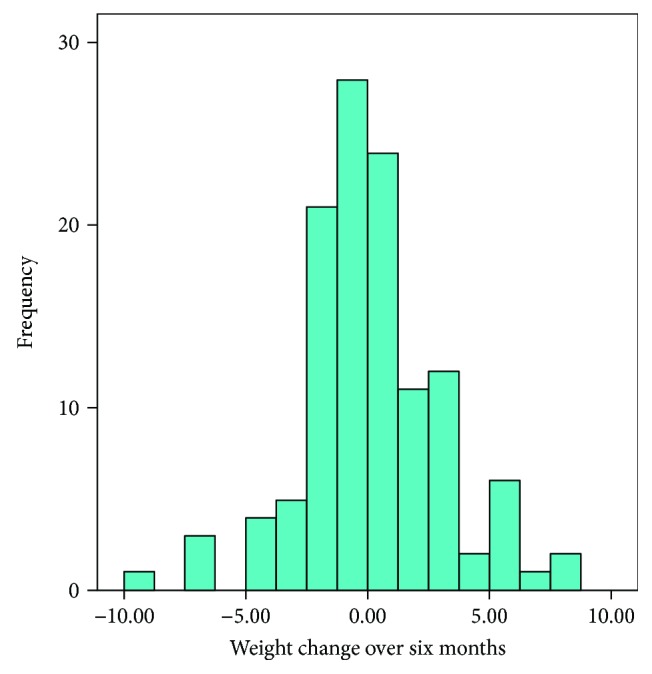
Histogram of weight change (kg) after 6 months in T2DM patients on lifestyle intervention (*n* = 120).

We reexamined data from a lifestyle intervention in people with T2DM [15, 16]; a randomized-control trial of 154 people with T2DM on the effect of cognitive behavioural treatment (CBT) (*n* = 76) versus control treatment (*n* = 78). The CBT consisted of three to six individual sessions of 30 minutes to increase the patient's motivation, by using principles of motivational interviewing, and ability to change their lifestyle, by using problem solving treatment. Although the overall weight loss in the CBT and control groups was averaged null (−0.05 kg) [15, 16], large intraindividual differences in weight changes were observed. About a third of the participants lost weight (−1 to −10 kg, *n* = 46), a third was weight stable (> −1 and <1 kg, *n* = 40), and a third gained weight (+1 to +15 kg, *n* = 39) after six months (see Figure 1). The Medical Ethical Committee of the VU University Medical Centre, Amsterdam, the Netherlands, approved the study design, protocols, information letters to the participants and informed consent form.

### 2.2. Population

The 154 people with T2DM were selected from the Hoorn Diabetes Care System cohort in West Friesland, the Netherlands. The Hoorn Diabetes Care System is a diabetes management programme, where people with T2DM annually visits the research centre for physical examination, information, and advice [17]. The selected participants fulfilled the following inclusion criteria: between 40 and 75 years old, able to understand the Dutch language, high risk for cardiovascular disease and diabetic complications. People with T2DM were considered at risk if they smoked and/or their body mass index (BMI) was ≥27.0 kg/m^2^ and/or their HbA1c-level was ≥52 mmol/mol (7.0%) [15, 16]. After six months, 6 participants discontinued the study (2 died, 3 refused to continue, 1 was treated for a severe disease), which leaves us with data of 148 participants of whom we have follow-up data. Of those 148 participants, 28 had missing data on eating traits at baseline or weight status at follow-up, leaving data of 120 participants for our current analysis. We observed no significant differences in age, sex, baseline weight, and HbA1c levels between participants who completed the study and those who dropped out or had missing data for eating traits or weight status (data not shown).

### 2.3. Measurements

We determined weight at baseline and after the 6-month intervention. Furthermore, at baseline, we determined eating traits as well as possible confounders.

#### 2.3.1. Eating Traits

Eating traits were assessed using the Dutch Eating Behaviour Questionnaire (DEBQ) [14]. This questionnaire consists of 33 items in two domains of eating traits, namely, restraint (10 items, including questions, e.g., “do you try to eat less at mealtimes than you would like to eat”) and disinhibition, measured as external eating (10 items, including questions, e.g., “if food tasted good, do you eat more than usual”), and emotional eating (13 items, including questions, e.g., “do you have desire to eat when you are irritated”). The items were scored on a five-point scale (1 = never, 3 = sometimes, and 5 = very often). Initial reliability and validity were provided by van Strien [12, 14]. The linear regression analysis of the eating trait score entered continuously showed an interaction effect. In line with this result and previous research on these (combination of) eating traits [18], we dichotomized the scores in the two domains based on median scores; restraint score > 3.0, external eating score > 2.2, and emotional eating score > 1.5. Participants were characterized as

- “successful dietary restrained” with high restraint and low disinhibition (restraint score > 3.0 combined with external eating score < 2.2 as well as emotional eating score < 1.5);
- “unsuccessful dietary restrained” with high restraint and high disinhibition (restraint score > 3.0 combined with external eating score > 2.2 or/and emotional eating score > 1.5);
- “low restrained” with low restraint scores (restraint score < 3.0) combined with any external eating and/or emotional eating scores.

The low restrained group was used as the reference group [14, 18].

#### 2.3.2. Weight Status

Weight (kg) and height (cm) were measured with participants wearing light clothes only, and the body mass index (BMI) was calculated as weight/height squared (kg/m^2^).

#### 2.3.3. Other Measures

Questionnaires were used to determine demographic and clinical variables, including sex, age (years), diabetes duration (years), and diabetes medication, stratified into the following groups: no medication, oral medication, or insulin users.

Blood was drawn when fasted to determine levels of plasma glucose (mmol/l) and HbA1c (mmol/l or %). Plasma glucose level was measured using the hexokinase method (Roche Diagnostics GmbH, Mannheim, Germany). HbA1c-levels were measured with high-performance liquid chromatography.

Additionally, questionnaires were used to assess lifestyle factors, including smoking status by asking participants if they were a current/past/no smoker as well as the Short Questionnaire to Assess Health-Enhancing (SQUASH) Physical Activity to determine physical activity, which was coded as a dichotomous variable, <30 minutes versus ≥ 30 minutes per day of physical activity.

Finally, depressive symptoms were determined using the Centre for Epidemiological Studies Depression (CES-D) scale. The overall score was calculated by summing up all scores, resulting in a score between 0 and 60. A score ≥ 16 implies depressive symptoms.

## 3. Statistical Analysis

First, normally distributed continuous data were presented as means (standard deviation) and skewed continuous data as medians (interquartile range). Second, we analysed the differences in the baseline characteristics between participants who completed the study and those who dropped out or had missing data for eating traits or weight status. Third, we analysed differences in the baseline characteristics for participants stratified for the three eating trait groups, using ANOVA or chi-square. Fourth, we used linear regression analysis to assess the association between combined eating trait groups and weight change after six months (model 1), with low restrained used as the reference group. We corrected for the following possible confounders: age and sex (model 2), insulin use (model 3), diabetes duration and levels of glucose and Hba1c (model 4), and physical inactivity, smoking and depressive symptoms (model 5). We determined confounders by assessing >10% in reported betas from model 1. Finally, we tested for an interaction with the CBT intervention, by adding CBT as an interaction term. Statistical analyses were performed with SPSS version 20.0 (SPSS Inc., Chicago, IL), and a two-sided *p* value below 0.05 was considered statistically significant.

## 4. Results

### 4.1. Participant Characteristics

The 120 people with T2DM included in our current analysis were on average 60.9 years of age at baseline and 62% was male. Their overall weight loss success was limited, with weight change ranging from −10 kg to +10 kg (see Figure 1), with a third of the people being weight stable, a third losing weight > −1 kg (average loss −2.6 ± 1.9 kg), and a third gaining weight > +1 kg (average gain +3.3 ± 1.9 kg). The characteristics of the participants, stratified for eating traits, are summarized in Table 1. We observed no differences between the eating trait groups in diabetes duration, insulin use, level of glucose and HbA1c, smoking, physical activity, or depression status. We did however observe that the successful restrained group were more often women and that the unsuccessful restrained were younger and had a higher BMI, compared to the respective other two groups.

With regard to association between eating traits and weight change after six months, we observed that when compared to the reference group (low restrained), the unsuccessful dietary restrained gained weight during the intervention, namely, 1.2 kg (confidence interval (CI) % = 0.1; 2), as shown in Table 2. No significant change was observed in the succesful dietary restrained group. With regard to possible confounding, the association was not confounded for the unsuccessful restrained group, while we did observe confounding for the successful restrained group and low restrained group. Finally, we observed no interaction effect (*p* = 0.7) with regard to the type of intervention received (CBT or control) as shown in Table 3.

## 5. Discussion

The aim of our study was to assess the association between eating traits and weight change after a lifestyle intervention in people with T2DM. First, we observed that weight loss success after our intervention was limited, with only a third of the participants losing weight. Despite the small variance, we did observe that eating traits explained variance in weight loss results, with those who at baseline were unsuccessful dietary restrained, that is, high restraint scores as well as disinhibition scores, gaining body weight during the six-month weight loss intervention, while the others were weight stable. Notwithstanding having the highest BMI at baseline and thus having the most weight to lose, the unsuccessful dietary restrained gained weight during the lifestyle intervention. To our knowledge, we are the first to investigate this association in people with T2DM.

Our results were comparable to studies in obese people without T2DM [6, 12, 13, 18], with those who were unsuccessful restrained being less able to lose weight and even gaining weight during the intervention. The group we studied was however metabolically very different, compared to earlier studies, having included people T2DM who were at high risk for cardiovascular disease and diabetic complications. This advanced disease status might explain the overall limited weight loss observed in our intervention and thereby explain the small absolute differences observed between eating traits in our current study. This small absolute difference is however a big relative difference with about a third of the weight gain explained by eating traits in our current study.

Overall, the results of our current study showed that eating traits are strongly associated with weight loss success (or rather no success) in people with T2DM who were subjected to a lifestyle intervention. This information could aid physicians to personalize weight-loss treatment. Assessing eating traits at baseline and subsequent choosing the right type of intervention could prevent foreseeable weight-loss failure and thus weight gain. Personalized weight-loss treatment for people with T2DM could include deterring from starting weight-loss treatment, but also psychological, pharmacological (i.e., GLP-1 antagonist), or surgical (bariatric surgery) treatment options [19]. Future studies should provide the scientific evidence for these options.

Our study has some limitations that need to be discussed. We only measured change in weight and not the change in mediating behaviours, such as physical activity or food intake. Second, we studied only a relative small population of people in an advanced disease state. This might explain the small overall change in weight and small variance, which makes it harder to detect possible associations. Future research should therefore be conducted in a larger population of people with T2DM and study long-term and mediating effects.

This study also has several strengths. First, we are the first to study these associations in people with T2DM, while up until now only overweight participants without T2DM were included [6, 12, 13]. Overall, our data contribute to the knowledge on weight change in people with T2DM and could provide us with possible new angels for weight-loss trials in this high-risk group of people with T2DM.

In conclusion, we observed that the eating trait of being unsuccessfully dietary restrained is associated with weight-loss failure after a six-month lifestyle intervention in people with T2DM. This information could aid physicians to personalize weight loss treatment.

## Figures and Tables

**Table 1:** Baseline characteristics of participants (*n* = 120).

	Low restrained (reference)	Successful dietary restrained	Unsuccessful dietary restrained	Overall *p* value
Number	56	12	52	
Men (%)	71%	33%	60%	0.04
Age (years)	62 ± 9	63 ± 8	58 ± 8	0.03
Body mass index (kg/m^2^)	30.1 ± 4	30.8 ± 3	33.7 ± 6	0.002
Diabetes duration (year)	8.3 ± 5	7.4 ± 5	6.6 ± 6	0.33
Diabetes medication (% insulin users)	33	17	23	0.31
Fasting plasma glucose (mmol/l)	7.8 ± 2	7.5 ± 1	7.8 ± 2	0.91
HbA1c-level (mmol/l)	6.7 ± 1	6.4 ± 1	6.8 ± 1	0.33
Smoking (current/past%)	21/54	17/42	26/47	0.77
Physical activity ≥ 30 min per day (%)	64	77	71	0.59
Depressive symptoms (% of CES-D score ≥ 16)	17	8	23	0.44

**Table 2:** The association between weight (kg) change stratified by eating behaviour in people with T2DM (*n* = 120).

Variable	Model 1	Model 2	Model 3	Model 4	Model 5
Low restrained (reference)	−0.7 (−1; 0.1)	−0.2 (−1; 1)	**−1.2 (−2; −0.3)**	−2.0 (−5; 2)	−0.5 (−2; 1)
Successful dietary restrained	0.01 (−2; 2)	0.2 (−2; 2)	0.3 (−2; 2)	0.1 (−2; 2)	0.1 (−2; 2)
Unsuccessful dietary restrained	**1.2 (0.1; 2)**	**1.3 (0.1; 2)**	**1.4 (0.3; 2)**	**1.2 (0.1; 2)**	**1.2 (0.1; 2)**

Presented betas and 95% confidence intervals. Bold: *p* < 0.05; Model 1: crude; Model 2: adjusted for age and sex; Model 3: adjusted for insulin use; Model 4: adjusted for diabetes duration and levels of glucose and Hba1c; Model 5: adjusted for physical inactivity, smoking, and depressive symptoms.

**Table 3:** The association between weight change (kg) by eating behaviour in people with T2DM stratified for intervention type (cognitive behavioural therapy, CBT *n* = 52) and control (*n* = 68).

Variable	CBT	Control
Low restrained (reference)	−0.8 (−2; 1)	−0.6 (−2; 1)
Successful dietary restrained	0.2 (−3; 3)	0.1 (−2; 2)
Unsuccessful dietary restrained	1.3 (−0.5; 3)	1.3 (−0.5; 3)

Presented betas and 95% confidence intervals.

## Data Availability

Data from this study are available for researchers who meet the criteria for access to confidential data. Please contact hoornstudy@vumc.nl to request access.

## References

[1] (1995). United Kingdom Prospective Diabetes Study (UKPDS) 13: Relative efficacy of randomly allocated diet, sulphonylurea, insulin, or metformin in patients with newly diagnosed non-insulin dependent diabetes followed for three years. *BMJ*.

[2] Anderson J. W., Kendall C. W. C., Jenkins D. J. A. (2003). Importance of weight management in type 2 diabetes: review with meta-analysis of clinical studies. *Journal of the American College of Nutrition*.

[3] Henry R. R., Chilton R., Garvey W. T. (2013). New options for the treatment of obesity and type 2 diabetes mellitus (narrative review). *Journal of Diabetes and Its Complications*.

[4] Campmans-Kuijpers M. J., Sluijs I., Nöthlings U. (2016). The association of substituting carbohydrates with total fat and different types of fatty acids with mortality and weight change among diabetes patients. *Clinical Nutrition*.

[5] Blak B. T., Rigney U., Sternhufvud C., Davis J., Hammar N. (2016). Weight change and healthcare resource use in English patients with type 2 diabetes mellitus initiating a new diabetes medication class. *International Journal of Clinical Practice*.

[6] Guaraldi F., Pagotto U., Pasquali R. (2011). Predictors of weight loss and maintenance in patients treated with antiobesity drugs. *Diabetes, Metabolic Syndrome and Obesity: Targets and Therapy*.

[7] Young M. D., Morgan P. J., Plotnikoff R. C., Callister R., Collins C. E. (2012). Effectiveness of male-only weight loss and weight loss maintenance interventions: a systematic review with meta-analysis. *Obesity Reviews*.

[8] Lih A., Pereira L., Bishay R. H. (2015). A novel multidisciplinary intervention for long-term weight loss and glycaemic control in obese patients with diabetes. *Journal of Diabetes Research*.

[9] Norris S. L., Zhang X., Avenell A. (2004). Long-term effectiveness of lifestyle and behavioral weight loss interventions in adults with type 2 diabetes: a meta-analysis. *The American Journal of Medicine*.

[10] Terranova C. O., Brakenridge C. L., Lawler S. P., Eakin E. G., Reeves M. M. (2015). Effectiveness of lifestyle-based weight loss interventions for adults with type 2 diabetes: a systematic review and meta-analysis. *Diabetes, Obesity and Metabolism*.

[11] Franz M. J., Boucher J. L., Rutten-Ramos S., VanWormer J. J. (2015). Lifestyle weight-loss intervention outcomes in overweight and obese adults with type 2 diabetes: a systematic review and meta-analysis of randomized clinical trials. *Journal of the Academy of Nutrition and Dietetics*.

[12] Vogels N., Westerterp-Plantenga M. S. (2007). Successful long-term weight maintenance: a 2-year follow-up. *Obesity*.

[13] Teixeira P. J., Carraça E. V., Marques M. M. (2015). Successful behavior change in obesity interventions in adults: a systematic review of self-regulation mediators. *BMC Medicine*.

[14] van Strien T. (1999). Success and failure in the measurement of restraint: notes and data. *The International Journal of Eating Disorders*.

[15] Welschen L. M. C., van Oppen P., Bot S. D. M., Kostense P. J., Dekker J. M., Nijpels G. (2013). Effects of a cognitive behavioural treatment in patients with type 2 diabetes when added to managed care; a randomised controlled trial. *Journal of Behavioral Medicine*.

[16] Welschen L. M. C., van Oppen P., Dekker J. M., Bouter L. M., Stalman W. A. B., Nijpels G. (2007). The effectiveness of adding cognitive behavioural therapy aimed at changing lifestyle to managed diabetes care for patients with type 2 diabetes: design of a randomised controlled trial. *BMC Public Health*.

[17] Walraven I., Mast M. R., Hoekstra T. (2015). Real-world evidence of suboptimal blood pressure control in patients with type 2 diabetes. *Journal of Hypertension*.

[18] Westerterp-Plantenga M. S. (2000). Eating behavior in humans, characterized by cumulative food intake curves—a review. *Neuroscience & Biobehavioral Reviews*.

[19] Van Gaal L., Scheen A. (2015). Weight management in type 2 diabetes: current and emerging approaches to treatment. *Diabetes Care*.

